# Elastic Energy Storage and Radial Forces in the Myofilament Lattice Depend on Sarcomere Length

**DOI:** 10.1371/journal.pcbi.1002770

**Published:** 2012-11-15

**Authors:** C. David Williams, Michael Regnier, Thomas L. Daniel

**Affiliations:** 1 Department of Physiology and Biophysics, University of Washington, Seattle, Washington, United States of America; 2 Department of Bioengineering, University of Washington, Seattle, Washington, United States of America; 3 Department of Biology, University of Washington, Seattle, Washington, United States of America; University of California San Diego, United States of America

## Abstract

We most often consider muscle as a motor generating force in the direction of shortening, but less often consider its roles as a spring or a brake. Here we develop a fully three-dimensional spatially explicit model of muscle to isolate the locations of forces and energies that are difficult to separate experimentally. We show the strain energy in the thick and thin filaments is less than one third the strain energy in attached cross-bridges. This result suggests the cross-bridges act as springs, storing energy within muscle in addition to generating the force which powers muscle. Comparing model estimates of energy consumed to elastic energy stored, we show that the ratio of these two properties changes with sarcomere length. The model predicts storage of a greater fraction of energy at short sarcomere lengths, suggesting a mechanism by which muscle function shifts as force production declines, from motor to spring. Additionally, we investigate the force that muscle produces in the radial or transverse direction, orthogonal to the direction of shortening. We confirm prior experimental estimates that place radial forces on the same order of magnitude as axial forces, although we find that radial forces and axial forces vary differently with changes in sarcomere length.

## Author Summary

Locomotion requires energy. Very fast locomotion requires a larger amount of energy than muscle can produce in such a short time period, thus muscle must use energy that it previously produced and stored as elastic deformation. Cyclical or repeated movements can be directly powered by muscle, but energy may be conserved in such cases through elastic energy storage. Traditionally we've looked primarily at tendons, insect exoskeletons, and bones as locations where this energy is stored. However, a small but growing body of literature has recently suggested the backbone filament proteins in muscle act as elastic storage locations. We suggest that the myosin motors themselves are capable of storing more energy than the filaments, energy that may be released to power very fast movements or reduce the cost of cyclical movements. We further suggest that this energy is stored in the radial deformations of myosin motors, in a direction that is perpendicular to the axis of muscle shortening.

## Introduction

### Energy storage in cross-bridges

Strain energy storage in muscle systems is most often associated with stretched tendons or other elastic supporting materials [1], [2]. In many instances, strain energy storage in skeletal and tendon structures has been shown to be a crucial component of the locomotor systems of animals, especially flying animals [3]. While muscle' role as a force generator has dominated research on animal locomotion, emerging studies posit diverse functional roles for muscles, including those of a brake, actuator, spring, or even a damper (for a review see Dickinson et al., 2000) [4]. Somewhat less attention has focused on the extent to which muscle itself plays a role in strain energy storage. That work which has been done has focused on the possibility of storing energy in the thick filaments, rigor cross-bridges, or the in the extensible accessory protein titin [5]–[7]. This assumption that active cross-bridges play a minor role is understandable: they generate force in activated muscle and are thought to be constantly cycling between freely diffusing and attached states and so would be expected to develop little deformation.

However, recent work suggests that in certain situations the cross-bridges may be locked onto muscle' thin filaments, frozen into a lattice that can act to store energy [8]. Energy storage may be possible in the subset of bound cross-bridges in antagonistic muscles that absorb inertial energy of a periodically moved appendage. This energy storage permits locomotion that would otherwise be energetically unfeasible [3]. Additionally, energy storage in muscle has been proposed in non-cyclical movements such as the tentacular strike of the squid, stomatopods'raptorial appendage strike, or the tongue extension of toads [9]–[12]. In these cases of one-off sudden movements, even a set of cycling cross-bridges may store strain energy for release on the initiation of rapid movement through pre-movement activation and subsequent pre-movement strain of the cross-bridges occurring just before the onset of an explosive motion. Our spatially explicit half-sarcomere model lets us parse how strain energy is partitioned between the filaments and the cross-bridges in maximally activated isometric sarcomeres. We show that the cross-bridges may store the majority of the elastic strain energy.

### Force generation in the radial direction

**Figure 1:**
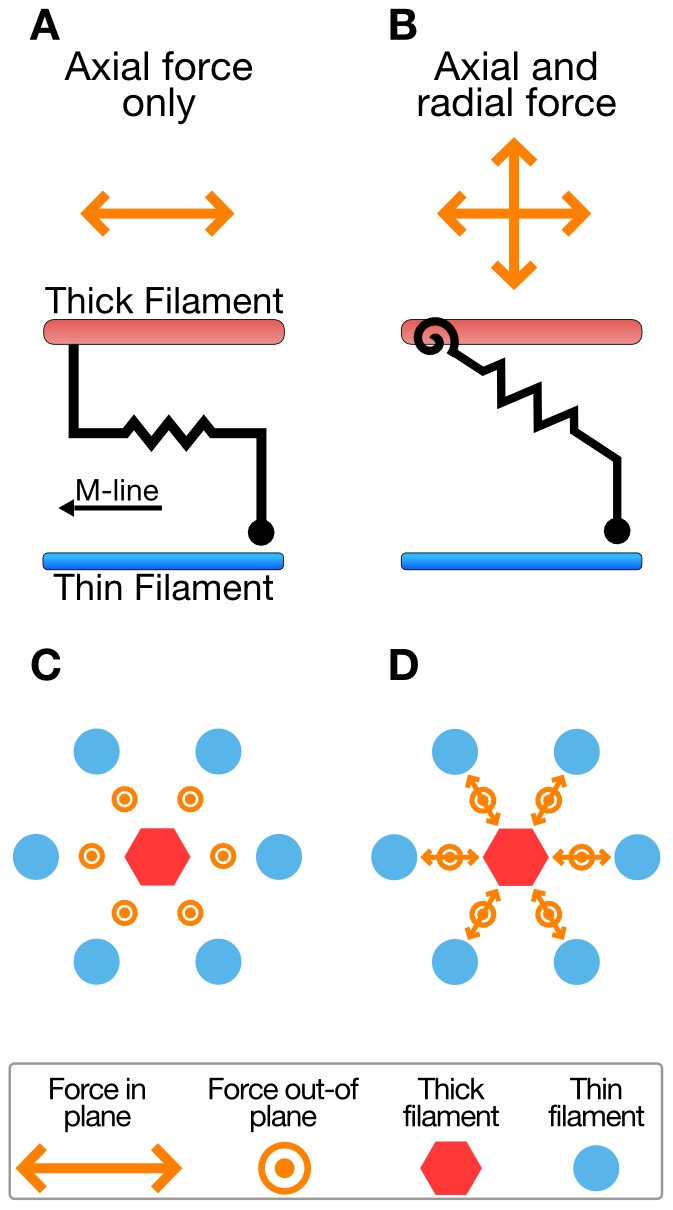
Models produce radial and axial forces. The one-dimensional cross-bridge model shown in (A) produces force and exists only in the axial direction. The two-dimensional cross-bridge model shown in (B) produces both axial and radial forces, and responds to changes in lattice spacing. A multi-filament model using one-dimensional cross-bridge, shown in (C), is diagrammed as a three-dimensional system but is insensitive to changes in lattice spacing and unable to explore radial force produced during contraction. Using two-dimensional cross-bridges in the same model geometry, in (D), allows the recording of radial forces and altered force dynamics with altered lattice spacing.

Cross-bridges are more often thought of as force generators than energy storage sites. The force generated by individual myosin heads arises from deformations as they form cross-bridges between the thick and thin filaments and undergo a rotation about a lever arm [13]. Interestingly, generating force by a rotation about a hinge implies that the vector of the generated force will have a component perpendicular to the direction of contraction [14], [15]. This force component is in the radial direction, orthogonal to the axial force that is generated in parallel to the thick and thin filaments (Figure 1).

Radial force was observed during contraction in intact muscle fiber experiments dating back to the 1950s [16]. Subsequent studies of radial forces placed them on the same order of magnitude as axial force [17]–[22]. These more recent experiments addressed radial force production through a proxy such as changes in fiber diameter or alterations of the muscle' radial compliance. The use of a lever-arm cross-bridge (with extensional and torsional springs) in the current spatially explicit model permits direct simulation of radial force production (Figure 1B&D) [15]. This cross-bridge model expands upon prior models (Figure 1A&C) [23]–[27].

Radial force may have functional implications. The internally generated radial force is a partial determinant of fiber radial compliance [21], [22]. Alterations in radial fiber compliance are also a hallmark of dystrophic disorders [28], [29]. Mis-regulation of the transmission of radial force produced during contraction may be a cause of the ultrastructural disorganization observed in histological studies of dystrophic muscle [30].

In addition to the more commonly analyzed axial forces, the model presented here addresses both radial force generation and the strain energy in the filaments and cross-bridges of the contractile lattice. These phenomena are linked, and are results of deformation of cross-bridges in the axial and radial directions. The interdependence of these properties is uniquely addressable using spatially explicit models of muscle contraction with lever-arm myosin geometries (Figure 1). We have developed such models based on protein structural information [15]. These models permit a fine parsing of energy locations which shows that cross-bridges store substantial elastic strain energy. Correlation of this cross-bridge energy with axial and radial forces suggests that radial cross-bridge strain could supply much of the energy stored in the contractile lattice.

## Results

Below we present results for simulations at the level of a half-sarcomere, the smallest fully-regulated component of muscle. Our half-sarcomere is composed of springs representing four myosin (thick) and eight actin (thin) filaments, arranged with boundary conditions which provide a semi-infinite lattice (Figure S1) [26]. Forces and energies are plotted as sarcomere length is changed by varying filament end locations. Spacing between the filaments is varied with sarcomere length to maintain a constant lattice volume [31]. Thus results are plotted over the range of the classic isovolumetric length-tension curve [32] The force decrease in our half-sarcomere model, to 18–21% of maximal values, at extreme sarcomere lengths is comparable to the 25–30% remaining force seen in experimental measurements of striated muscle [32].

Radial forces produced within the half sarcomere are both large and correlated with energy storage. Our model monitors radial forces produced by lever arm cross-bridge models composed of an angular (or torsional) and an extensional (linear) spring (Figure 1A&B). The forces and energies used for comparison are steady state values produced on full isometric activation at a range of sarcomere lengths stretching across the length-tension curve. Even though isometric contraction represents only one possible loading regime, it is a computationally simple condition with low cross-bridge turnover, making it a regime well suited to explore how sarcomere length, and thus filament overlap, influences force produced and energy stored.

### Axial and radial forces are of the same order of magnitude

**Figure 2:**
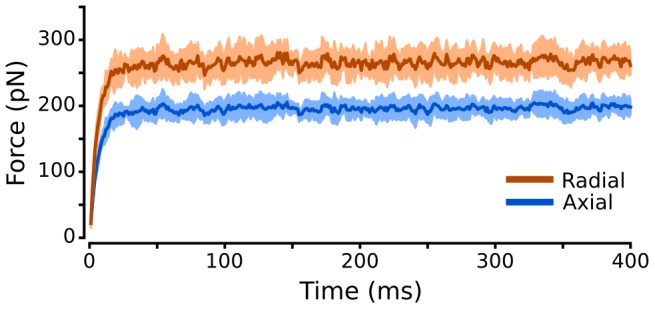
Example axial and radial forces. The mean (lines) and standard deviations (shaded regions) of axial and radial forces as they develop at a sarcomere length of 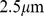 over the course of 10 runs. Each run consists of 400 time steps, each 1 ms long. Maximum forces are calculated from the mean of the last 50 ms of such runs.

In the fully activated conditions of our simulations, both the axial and radial forces quickly rise to an asymptotic maximum (Figure 2). This rise to a maximum value takes less than 50 ms. The exponential time constant of the rise to peak force is not significantly different between the axial and radial forces (p = 0.31). After steady force levels are reached, stochastic fluctuations in the number and states of bound cross-bridges show as noise in the force traces. This clean rise gives clear asymptotic maximum forces.

**Figure 3:**
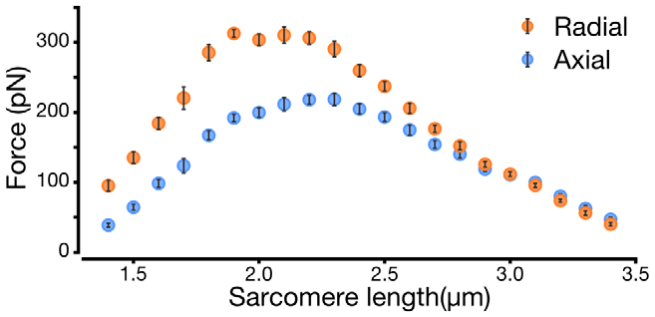
Radial force is of the same order of magnitude as axial force. Asymptotic maxima of 10 runs at each sarcomere length with standard deviation. Radial and axial forces obey similar scaling trends across the sarcomere lengths and lattice spacings of a classic length-tension curve. The level of radial force varies from 2.4 times the level of axial force at extremely short sarcomere lengths to 0.9 times the axial force at the longest sarcomere lengths. The radial force plateau ends at a shorter sarcomere length than does axial force plateau.

The radial force, at all sarcomere lengths, is of the same order of magnitude as the axial force (Figure 3). At most sarcomere lengths below 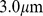, where there is significant myofilament overlap, the radial force is larger than the axial force. At very short sarcomere lengths the radial force is as much as 2.4 times the axial force, although this is in a region where overall axial force levels are relatively small. These results agree with prior experimental studies which found radial forces of the same magnitude as axial forces [18]–[22], [33].

### The cross-bridges store the majority of strain induced energy

**Figure 4:**
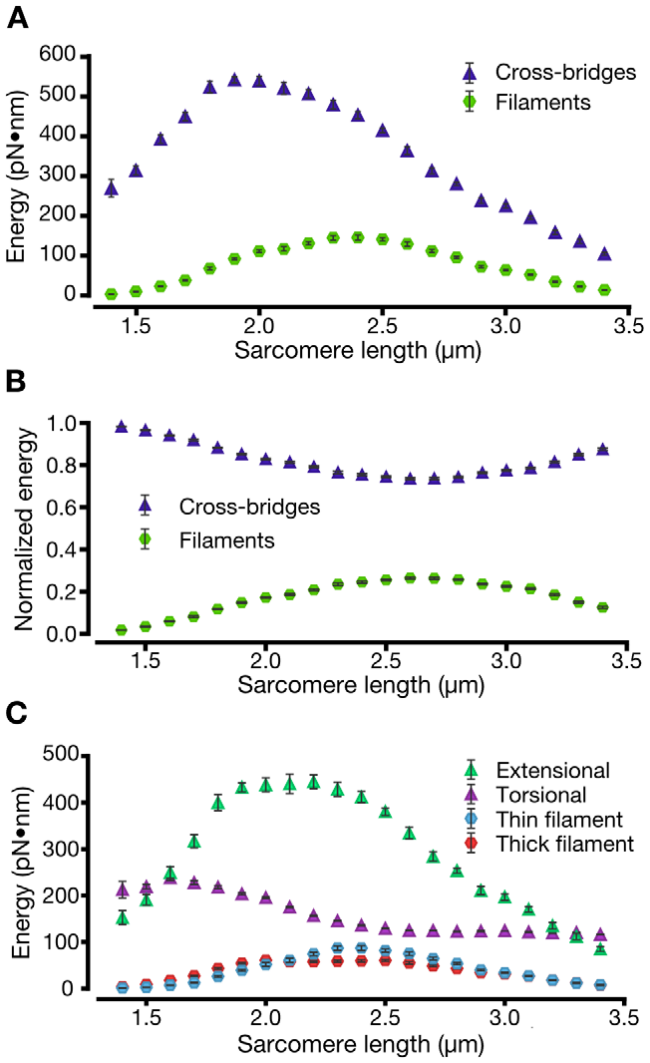
Energy of the multi-filament array is partitioned between the filament backbone and the cross-bridges. The energy stored in the springs comprising the cross-bridges and filaments changes, much as force does, with sarcomere length. (A) As sarcomere length increases, the energy stored across all cross-bridges rises and falls more steeply than does the energy stored in the filaments. (B) At all lengths, the energy stored in the cross-bridges comprises more than 3/4 of the sarcomere's strain based energy. (C) The energy stored in the thick and thin filaments is approximately equal, while the extensional spring of the cross-bridges stores the major share of the energy at all sarcomere lengths.

The majority of strain energy stored in the complete contractile lattice of filaments and cross-bridges is partitioned in the cross-bridges (Figure 4A&B). The relative distribution of energy between the cross-bridges and the filaments varies with sarcomere length (Figure 4B). Even when the filaments reach their peak energy relative to the cross-bridges, at the sarcomere lengths where maximum force is produced, the cross-bridges still have more than three times the energy of the filaments. At very long and very short sarcomere lengths, where little axial force is produced, the energy stored in the cross-bridges dominates the system.

The elastic energy storage may be more finely parsed: into the components located in each of the two springs constituting every cross-bridge and the components in each of the two filament types (Figure 4C). These results show that the energies of the thick and thin filaments vary similarly across all sarcomere lengths. In contrast, the energies of the torsional and extensional spring which comprise the cross-bridge are quite different. The energy of the torsional springs is far less than that of the extensional springs at all but the smallest sarcomere lengths. Thus the majority of the elastic strain felt by the cross-bridge may arise from stretching, rather than rotation.

### Cross-bridge energy correlates with radial force

Energy stored in the cross-bridges follows the radial force produced by the system (Figures 3&4A). Radial force has a higher correlation with the cross-bridges'energy than does axial force (linear fits show respective 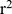 values of 0.97 and 0.69). All of these relationships are significant (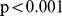). This suggests that radial strain in the system may disproportionately determine the energy stored in the cross-bridges or, put another way, radial deformation may be acting as a “idden”energy sink.

### Fractional energy stored is not constant

**Figure 5:**
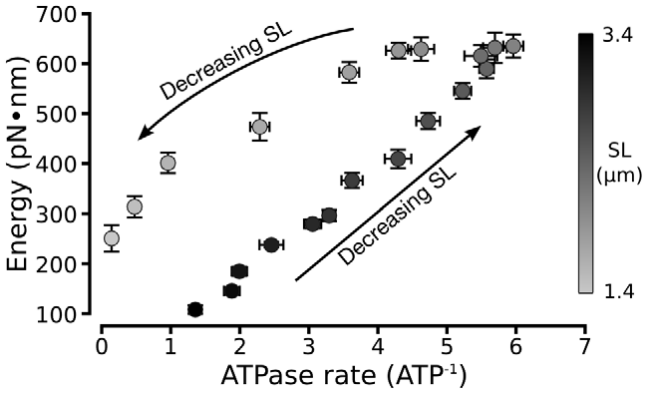
Energy stored varies with sarcomere length as well as energy input. All energy present in the isometrically contracting half-sarcomere derives from the hydrolysis of ATP. This permits a direct comparison of the energy input to the system, as measured by the consumption of ATP, to the energy stored across all filaments and cross-bridges. The fraction of energy stored is shown to change as sarcomere length drops below 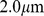. A contractile lattice with a stored energy dependent only on the rate at which ATP is consumed would not exhibit the hysteresis present as sarcomere length changes. This suggests that of the energy released by ATP, the fraction which is stored instead of being dissipated is partially determined by sarcomere length.

As the sarcomere shortens below 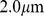, the ratio of energy stored in the sarcomere to energy consumed through ATP use is elevated (Figure 5). This fraction of energy stored, or energy retention efficiency, is constant at sarcomere lengths longer than 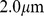. The hydrolysis of ATP to ADP alters the free energy of a modeled cross-bridge by 8.8 RT (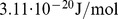) [15], [26]. A fraction of this energy is stored as continued deformation of the cross-bridge in its new state and a fraction drives deformation of the filaments experiencing cross-bridge forces. This energy is entirely released on the detachment of the cross-bridge and may partially appear as deformations induced in other bound cross-bridges. At sarcomere lengths longer than 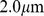 the ratio of the energy stored by the sarcomere to the power consumed by the sarcomere (as measured by the rate of ATP consumption) is constant (Figure 5). At shorter sarcomere lengths this ratio climbs: more of the input energy is stored in the sarcomere.

## Discussion

The role that muscle' radial geometry plays in determining its functioning is still poorly understood. It is difficult to experimentally measure the forces muscle generates in the radial direction and the strain and energies which result from such forces. The studies that have attempted to measure radial forces have all done so indirectly, through back-calculating from changes in radial stiffness or lattice spacing changes on activation [19], [20], [22]. While these are easier values to quantify, they are not direct measurements of radial force. Our results suggest radial forces, in addition to being quite large, may function as a “idden” energy storage mechanism, that radial force may partition energy into the cross-bridges which is not initially transmitted to the filament ends.

### Substantial energy is stored in muscle's contractile elements

The elements of the sacomere's contractile lattice, cross-bridges as well as thick and filaments, are storing a substantial amount of energy. At peak energy levels where the 16% of bound cross-bridges in our model store 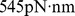 of energy, each bound cross-bridge is storing approximately 20% of the work it is capable of producing across a power stroke [34]. The peak cross-bridge energy levels we see in of our half-sarcomere model is 0.084 J/kg of stored energy when the lattice is assumed to have the density of water [31]. This is more than 10% of the flight-permitting energy stored in Hemipteran flight muscle [35].

This strain energy is primarily stored in the cross-bridges—rather than in the thick and thin filaments—despite the turnover and energy dissipation inherent in the our model of cross-bridge kinetics [15]. Energy storage in the cross-bridges requires low cross-bridge turnover, as the deformation of an individual cross-bridge, and thus the energy in an individual cross-bridge, is released upon detachment. A “locked lattice” of tightly bound cross-bridges is likely to be present in a maximally activated isometric contraction, as simulated here, and where external factors such as temperature differentials reduce cross-bridge turnover [8]. Energy stored in the muscle's filaments and cross-bridges is then available for later release and utilization.

### Radial force may be a ‘hidden’means of storing energy

Radial force' role in muscle remains unclear. Radial force may simply be a byproduct of the motor and filament geometry which has evolved to generate force or it may produce a useful effect. The high correlation between radial force and strain energy stored in the cross-bridges may indicate that radial force and distortion act as an energy storage mechanism which permits the cross-bridges to store more strain based energy than the thick and thin filaments. It is possible that radially associated energy could then be redirected to produce axial force, much as happens when energy is stored in the deformation of elastic solids. Such a mechanism would provide a means to store the energy powering after-stretch transient shortening, the shortening of recently stretched muscle against a load equal to its maximal isometric force [36]. Transient shortening has been suggested to be a result of energy elastically stored in cross-bridges at levels comparable to those seen in Figure 4 [34].

However, radial strain based energy storage will not necessarily register as force at the filament ends. As such, it may be difficult to address in experiments, although radial stiffness observations suggest a means by which such tension and energy storage could be quantified [22].

### The fraction of energy stored varies with muscle length

The variable energy retention efficiencies shown in Figure 5 represent a potential mechanism by which sarcomere parameters can determine a muscle' functional role, e.g. motor, brake, or spring. A muscle that stores little of its consumed energy and converts most into force acts as a motor, while a muscle that stores more of its consumed energy for later release is acting, at least prior to use of stored energy, as a spring. There are analogous selections between roles in lengthening and shortening muscle, as contrasted to the isometric conditions simulated here [4].

The non-constant storage of input energy means that changing the degree of filament overlap or lattice spacing affects the amount of energy stored in muscle and thus the amount of energy which may be released to power contraction. Thus it is possible that operating at different sarcomere lengths could change the efficiency with which muscle retains energy or dissipates it, driving the muscle towards functioning as a spring or a break.

Energy storage in muscle is still a relatively unexplored field. The highly structured and three-dimensional nature of muscle makes it likely that, historically, we have overlooked forms of energy storage and efficiency regulation. This work points towards cross-bridges as a site where energy can be stored, either in preparation for use in rapid movements or to reduce the energy requirements of cyclical movements. Further investigation of how energy is partitioned between sub-sarcomeric structures and of how radial force is generated will continue to expand our understanding of these new mechanisms. Particularly, this work may help us to understand cases where energy is stored as deformations in an axis orthogonal to that of the direction of muscle shortening, such as in a proposed mechanism by which the heart stores elastic strain introduced into transverse fibers during filling [37].

## Methods

Our spatially-explicit model of the half-sarcomere represents the thick and thin filaments as chains of springs connecting each myosin crown or actin-binding site [25], [26]. The cross-bridges linking the contractile filaments are two-dimensional, and thus both produce radial force and are sensitive to changes in lattice spacing [15]. This is the only model we are aware of that accounts for the lattice spacing between filaments and employs a cross-bridge capable of simulating force in the radial direction.

### Thick/thin filament arrangement and geometry

Four thick and eight thin filaments are arranged in an evenly spaced hexagonal lattice with toroidal boundary conditions. As shown in Figure S1, this filament arrangement simulates an infinite lattice [26]. The filament numbers and arrangement of boundary conditions provides the smallest system in which no single thick filament connects to two sides of a single thin filament, and vice versa. The distance between the faces of these filaments, here referred to as the lattice spacing, is uniform and used to provide the distance across which myosin must diffuse in order to bind [15].

Lattice spacing changes with sarcomere length to maintain a constant lattice volume. Thus lattice spacing separating the faces of adjacent thick and thin filaments (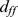) is given from sarcomere length (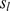) by 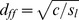 where 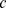 is a proportionality constant. This proportionality constant (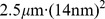) is chosen to set the lattice spacing to 14 nm at a sarcomere length of 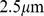, values consistent with a wide range of muscle types [31].

Along each thick filament are 60 myosin crowns, with three myosin heads per crown. The myosin heads on a given crown are azimuthally rotated by 120 degrees from their neighbors. The crowns are grouped into a three crown, 43 nm repeating pattern [26]. Progressing axially through the pattern, each crown is azimuthally rotated relative to the prior crown by 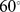. This rotation pattern is measured and described in Al-Khayat et al., 2008 [38]. As a result of this crown rotation every myosin head faces an opposite a thin filament with which it may interact.

Each thin filament is made up of two actin strands. Each strand hosts 45 actin binding-sites giving a whole filament 90 actin binding-sites [25], [26], [39]. Each binding site faces and interacts with one of three adjacent thick filaments. Consecutive binding sites on each strand are rotated by 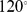 clockwise. The first binding site of one strand is offset from the first binding site of the other strand by half the axial distance between adjacent binding sites and a rotation of 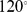 counter-clockwise. Further filament properties are listed in Table S1.

### Cross-bridge model, briefly

Our cross-bridges are comprised of one torsional spring and one extensional spring [15]. The axial and radial location of each myosin tip determines the angle and extension of the cross-bridge springs and thus the force the cross-bridge generates. The torsional spring simulates the power stroke via a change in rest angle.

Inefficiency in converting, through ATP hydrolysis, chemical to mechanical energy during state transitions is accounted for as distortions of the cross-bridge. This inefficiency is manifest as heat. Additionally as we suggest below, mechanical strain energy which drives motion may also be returned as recoil of cross-bridges or filament backbones.

The binding of an individual myosin head is determined by the distance to the nearest available binding site and energy landscape created by the properties of the head' constituent springs. The process is one of perturbation, distance calculation, and stochastic attachment. A myosin head is perturbed with a random Boltzmann distributed energy, providing a new myosin tip location [40]. Distance from the myosin tip to the nearest available binding site is calculated. Binding probability, which falls off exponentially as the distance to the binding site increases, is checked against a random number. Further transitions between loosely attached, force generating, and unattached states are determined as described in Williams et al., 2010 [15].

### Force transmission through the lattice - calculation of axial and radial forces

The thick and thin filaments are coupled together by the cross-bridges. Each bound cross-bridge both generates and transmits force. This coupling yields a three dimensional network of springs.

We solve for the root location of our spring-network at each time-step. The root is the set of locations of actin binding-sites and myosin crowns that provides no net axial force at any internal point in the spring-network. A modified form of the Powell hybrid method allows the actin and myosin locations to iteratively settle into their solution values [41].

At each time-step, actin and myosin locations are allowed to settle in the axial dimension while being held rigidly in the radial dimension. The total axial force (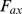) of the system thus comes as the sum of unbalanced axial forces at the ends of each thick filament,
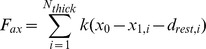
where for a given thick filament, 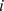, 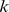 is the filament stiffness, 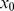 is the axial location of the end node location, 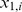 is the axial location of the adjacent node, and 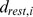 is the resting separation between the two. The total radial force (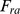) is the sum of the radial force experienced by all sides of the thick filament and thus ultimately the sum of the radial force of each cross-bridge (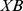),
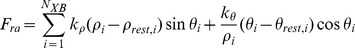
where 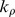, 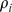, and 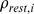 are the stiffness, length, and rest length of cross-bridge 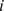
's extensional spring while 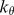, 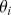, and 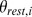 are the stiffness, angle, and rest angle of cross-bridge 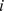
's torsional spring. The current model does not permit radial movement as it does not include a resistive radial force, i.e. forces in the radial direction that act against the radial deformation of the filament.

Future models may treat the filaments as radially-deformable axially-tensioned beams subject to filament persistence length, electrostatic effects, and viscous stresses and thus be able to permit radial movement. Radial bending or deformation of the thick and thin filaments could potentially reduce the level of radial force within the lattice by increasing lattice spacing disorder. Reduced radial force has the potential to affect the partitioning of energy between the cross-bridges and filaments, shifting energy stored in cross-bridge deformation to a newly-created radial deformation component of the thick and thin filaments' energies. Radial bending of the filaments and subsequent changes in the distribution of axial and radial forces will be resisted by the highly constrained nature of the sarcomere lattice as well as the inherent stiffnesses of the filaments themselves.

### Energies of a filament or cross-bridge is the sum of its springs'energies

The energy in a cross-bridge or filament is the sum of the energy in every spring in that cross-bridge or filament. Thus the energy of a single cross-bridge (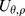) is calculated, as in prior work [15].
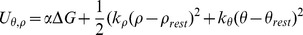
The energy of a thick filament (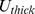) with 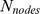 crown locations is calculated as
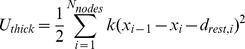
and the energy of a thin filament is calculated similarly. These energies are logged throughout the run of an instance of the model and their stable state is found at the conclusion of the simulation.

### Simulation details

A simulated contraction follows the course described in the diagram shown in Figure S2. Briefly, each 1 ms time-step consists of allowing every myosin head to calculate the probability of changing from its current state into another state, check this probability against a random number, and transition or not based on the outcome. After the state of each myosin head has been established, the location of every interior point in the model is allowed to settle so that there is no net axial force on them. The axial force, radial force, and other properties of the system at that time-step are then recorded and a new time-step is begun if the contraction has not yet reached its end.

The model was allowed to complete 10 contractions (starting from unbound cross-bridges) for every set of input parameters, each continuing for 400 ms (400 time-steps at 1 ms resolution). The asymptotically developed forces and energies were calculated as the mean of the force produced over the last 50 ms.

These simulations took place on a dynamically created cluster of spot-priced machine instances in Amazon's EC2 service (Figure S3). Control of this cluster was with a first-in-first-out command queue hosted by Amazon's SQS.

## Supporting Information

Figure S1 **Model lattice arrangement.** The model simulates a semi-infinite lattice with four myosin and eight actin filaments, as in Tanner et al., 2007. The bolder filaments and cross-bridge interactions are those which are directly simulated, while the desaturated filaments are the bold filaments mirrored across a boundary. Cross-bridge interactions that cross a boundary condition to a mirrored thin filament are connected only to their non-mirrored thick filament. This lattice geometry is used as it is the smallest arrangement of thick and thin filaments that: 1) maintains the physiological ratio of thick to thin filaments and 2) permits tessellation of the existing filaments without causing a single thick filament to face a thin filament more than once.
(PDF)

Figure S2 **Model code structure and information flow.** A diagrammatic representation of the steps that occur during a simulation, and which produce the measured forces and energies.
(PDF)

Figure S3 **Sequence diagram of remote simulation process.** This sequence depicts the process of running and retrieving results from Amazon's Web Services (AWS). Three AWS services are used: the Simple Storage Service (S3), the Simple Queue Service (SQS), and the Elastic Compute Cloud (EC2). The custom Python code which constitutes the model is sent to and stored in S3. The parameters which describe simulations are parsed into small jobs, which are sent to SQS. The remote cluster which will run the simulation is configured on EC2. When machine instances which make up the cluster are allocated and have started, they connect to S3 and download a copy of the model. They then connect to SQS and request jobs to run on each of their available cores. As jobs are completed, their results are uploaded to S3 and the completed job is removed from the SQS queue. The machine instances of the cluster will continue this process until the job queue is empty at which point they shut themselves down. The complete result set may then be downloaded for local processing, or processed by another EC2 cluster, as needed.
(PDF)

Table S1 **Geometric and mechanical properties.** A listing of the geometric and mechanical parameters used in the model. Where possible, values used are common to a wide array of striated muscle. By choosing conserved values, a more general model is produced and the necessity of computationally intractable sensitivity analyses is avoided. All values are given for the half-sarcomere model and thus refer to one half of a thick or a thin filament.
(PDF)
