# Supplementary material for: Elastic Energy Storage and Radial Forces in the Myofilament Lattice Depend on Sarcomere Length
Source: PLoS Comput Biol. 2012 Nov 15;8(11):e1002770. doi: 10.1371/journal.pcbi.1002770 (PMC3499250; doi:10.1371/journal.pcbi.1002770)
Supplement: Figure S1 — Model lattice arrangement. The model simulates a semi-infinite lattice with four myosin and eight actin filaments, as in Tanner et al., 2007. The bolder filaments and cross-bridge interactions are those which are directly simulated, while the desaturated filaments are the bold filaments mirrored across a boundary. Cross-bridge interactions that cross a boundary condition to a mirrored thin filament are connected only to their non-mirrored thick filament. This lattice geometry is used as it is the smallest arrangement of thick and thin filaments that: 1) maintains the physiological ratio of thick to thin filaments and 2) permits tessellation of the existing filaments without causing a single thick filament to face a thin filament more than once. (PDF) [file pcbi.1002770.s001.pdf]

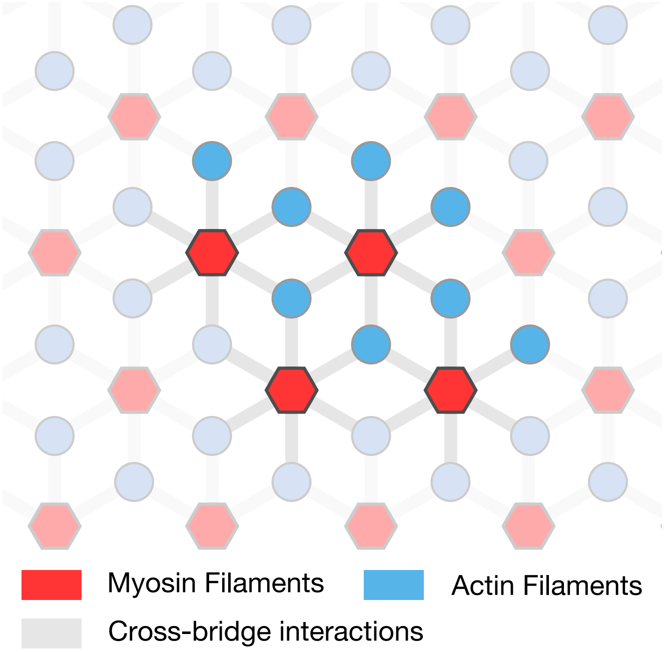

Figure S1: Model lattice arrangement. The model simulates a semi-infinite lattice with four myosin and eight actin filaments, as in Tanner et al., 2007. The bolder filaments and cross-bridge interactions are those which are directly simulated, while the desaturated filaments are the bold filaments mirrored across a boundary. Cross-bridge interactions that cross a boundary condition to a mirrored thin filament are connected only to their non-mirrored thick filament. This lattice geometry is used as it is the smallest arrangement of thick and thin filaments that: 1) maintains the physiological ratio of thick to thin filaments and 2) permits tessellation of the existing filaments without causing a single thick filament to face a thin filament more than once.
