# Supplementary material for: Elastic Energy Storage and Radial Forces in the Myofilament Lattice Depend on Sarcomere Length
Source: PLoS Comput Biol. 2012 Nov 15;8(11):e1002770. doi: 10.1371/journal.pcbi.1002770 (PMC3499250; doi:10.1371/journal.pcbi.1002770)
Supplement: Figure S2 — Model code structure and information flow. A diagrammatic representation of the steps that occur during a simulation, and which produce the measured forces and energies. (PDF) [file pcbi.1002770.s002.pdf]

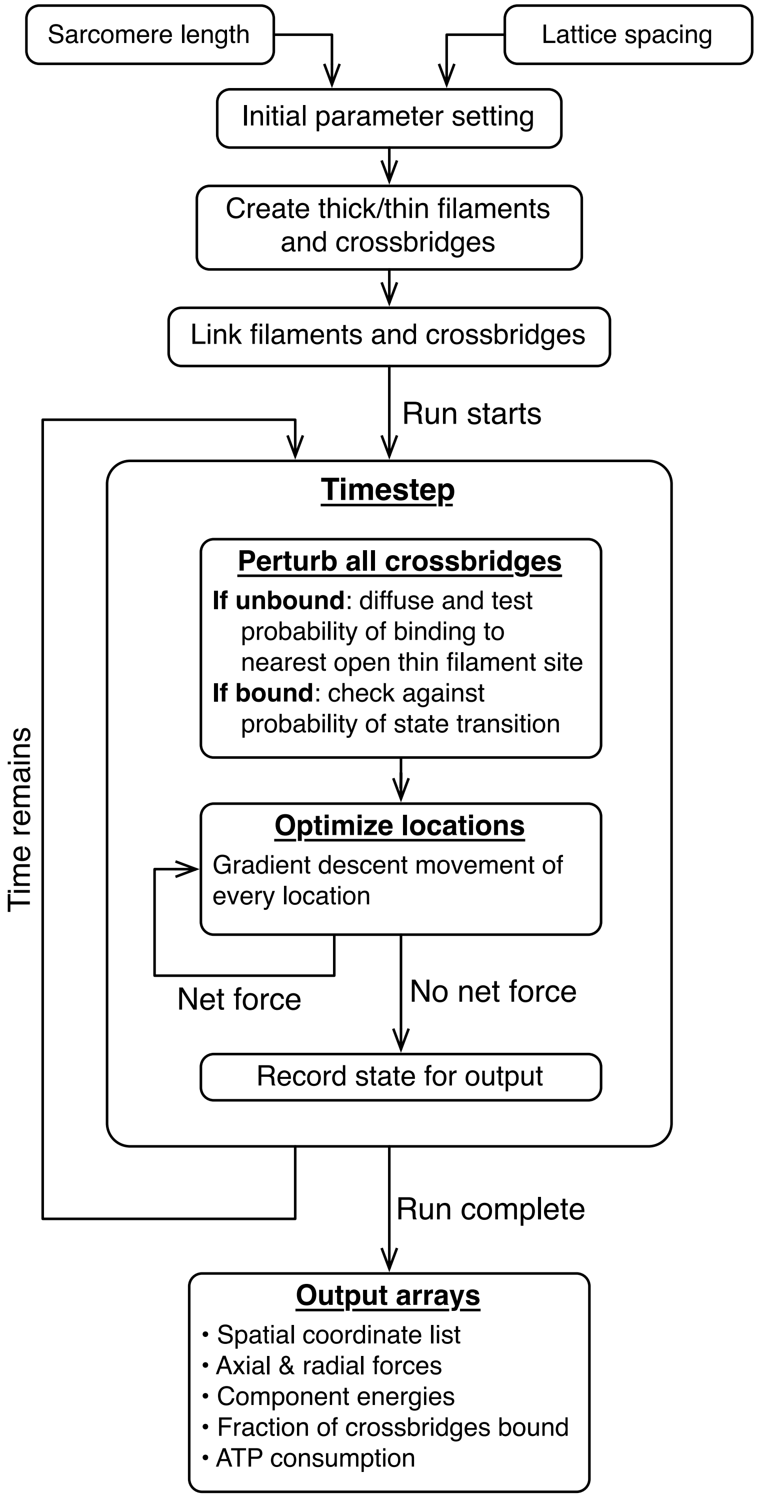

Figure S2: Model code structure and information flow. A diagrammatic representation of the steps that occur during a simulation, and which produce the measured forces and energies.
