# Supplementary material for: Elastic Energy Storage and Radial Forces in the Myofilament Lattice Depend on Sarcomere Length
Source: PLoS Comput Biol. 2012 Nov 15;8(11):e1002770. doi: 10.1371/journal.pcbi.1002770 (PMC3499250; doi:10.1371/journal.pcbi.1002770)
Supplement: Figure S3 — Sequence diagram of remote simulation process. This sequence depicts the process of running and retrieving results from Amazon's Web Services (AWS). Three AWS services are used: the Simple Storage Service (S3), the Simple Queue Service (SQS), and the Elastic Compute Cloud (EC2). The custom Python code which constitutes the model is sent to and stored in S3. The parameters which describe simulations are parsed into small jobs, which are sent to SQS. The remote cluster which will run the simulation is configured on EC2. When machine instances which make up the cluster are allocated and have started, they connect to S3 and download a copy of the model. They then connect to SQS and request jobs to run on each of their available cores. As jobs are completed, their results are uploaded to S3 and the completed job is removed from the SQS queue. The machine instances of the cluster will continue this process until the job queue is empty at which point they shut themselves down. The complete result set may then be downloaded for local processing, or processed by another EC2 cluster, as needed. (PDF) [file pcbi.1002770.s003.pdf]

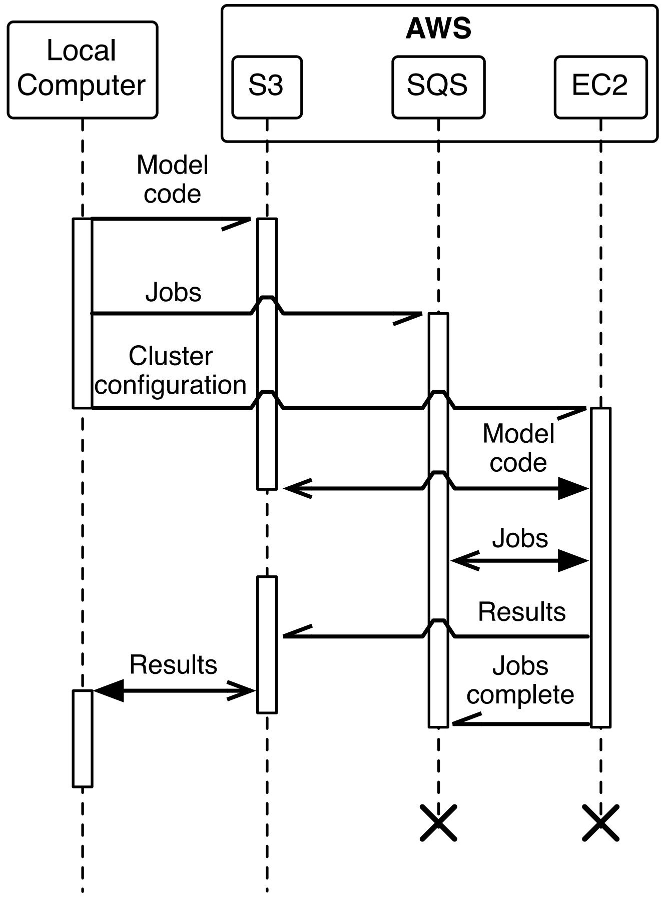

Figure S3: Sequence diagram of remote simulation process. This sequence depicts the process of running and retrieving results from Amazon's Web Services (AWS). Three AWS services are used: the Simple Storage Service (S3), the Simple Queue Service (SQS), and the Elastic Compute Cloud (EC2). The custom Python code which constitutes the model is sent to and stored in S3. The parameters which describe simulations are parsed into small jobs, which are sent to SQS. The remote cluster which will run the simulation is configured on EC2. When machine instances which make up the cluster are allocated and have started, they connect to S3 and download a copy of the model. They then connect to SQS and request jobs to run on each of their available cores. As jobs are completed, their results are uploaded to S3 and the completed job is removed from the SQS queue. The machine instances of the cluster will continue this process until the job queue is empty at which point they shut themselves down. The complete result set may then be downloaded for local processing, or processed by another EC2 cluster, as needed.
