# Supplementary material for: Elastic Energy Storage and Radial Forces in the Myofilament Lattice Depend on Sarcomere Length
Source: PLoS Comput Biol. 2012 Nov 15;8(11):e1002770. doi: 10.1371/journal.pcbi.1002770 (PMC3499250; doi:10.1371/journal.pcbi.1002770)
Supplement: Table S1 — Geometric and mechanical properties. A listing of the geometric and mechanical parameters used in the model. Where possible, values used are common to a wide array of striated muscle. By choosing conserved values, a more general model is produced and the necessity of computationally intractable sensitivity analyses is avoided. All values are given for the half-sarcomere model and thus refer to one half of a thick or a thin filament. (PDF) [file pcbi.1002770.s004.pdf]

Table S1 - Geometric and mechanical properties

Filament half	Parameter	Value	Source
Thick filament	Rest length	858 nm	[1]
	Undecorated region length	80 nm	[2]
	Inter-crown spacing	14.3 nm	[1]
	Number of crowns	60 /filament	[1]
	Compliance	2020 pN/nm	[3]
Thin filament	Rest length	1119 nm	[1]
	Binding-site spacing	12.4 nm	[1]
	Rotation between sites	240°	[1]
	Compliance	1743 pN/nm	[3]

A listing of the geometric and mechanical parameters used in the model. Where possible, values used are common to a wide array of striated muscle. By choosing conserved values, a more general model is produced and the necessity of computationally intractable sensitivity analyses is avoided. All values are given for the half-sarcomere model and thus refer to one half of a thick or a thin filament.

Sources are as follows:

1. Tanner BCW, Daniel TL, Regnier M (2007) Sarcomere lattice geometry influences cooperative myosin binding in muscle. PLoS Comput Biol 3: e115.

2. Higuchi H, Yanagida T, Goldman YE (1995) Compliance of thin filaments in skinned fibers of rabbit skeletal muscle. Biophys J 69: 1000–10

3. Daniel TL, Trimble AC, Chase PB (1998) Compliant realignment of binding sites in muscle: transient behavior and mechanical tuning. Biophys J 74: 1611–21.
